# Intriguing Cytotoxicity of the Street Dissociative Anesthetic Methoxphenidine: Unexpected Impurities Spotted

**DOI:** 10.3390/ijms23042083

**Published:** 2022-02-14

**Authors:** Bronislav Jurásek, Silvie Rimpelová, Martin Babor, Jan Čejka, Vilém Bartůněk, Martin Kuchař

**Affiliations:** 1 Forensic Laboratory of Biologically Active Substances, Department of Chemistry of Natural Compounds, University of Chemistry and Technology, Prague, Technická 5, 166 28 Prague, Czech Republic; bronislav.jurasek@vscht.cz; 2 Department of Experimental Neurobiology, National Institute of Mental Health, Topolová 748, 250 67 Klecany, Czech Republic; 3 Department of Biochemistry and Microbiology, University of Chemistry and Technology, Prague, Technická 5, 166 28 Prague, Czech Republic; 4 Department of Solid State Chemistry, University of Chemistry and Technology, Prague, Technická 5, 166 28 Prague, Czech Republic; martin.babor@vscht.cz (M.B.); jan.cejka@vscht.cz (J.Č.); 5 Department of Inorganic Chemistry, University of Chemistry and Technology, Prague, Technická 5, 166 28 Prague, Czech Republic; vilem.bartunek@vscht.cz

**Keywords:** crystal structure, cytotoxicity, dissociative anesthetic, inorganic impurity, methoxphenidine, novel synthetic drug, new psychoactive substances, X-ray powder diffraction

## Abstract

The black market for new psychoactive substances has been constantly evolving and the substances that appear on this market cause a considerable number of issues, in extreme cases leading to human deaths. While monitoring the drug black market, we detected a sample of a dissociative anesthetic methoxphenidine, the salt of which contained an unusual anion in the form of bromo- and chloro-zincate complex. Concerning the unknown and potentially hazardous properties of this sample, we performed an in vitro cytotoxicity screening in cell lines of various origins (e.g., kidney, liver, bladder) which was compared with the toxicity results of the methoxphenidine standard prepared for this purpose. The street methoxphenidine sample exhibited markedly higher toxicity than the standard, which was probably caused by the anion impurity. Since it is not usual to analyze anions in salts of novel psychoactive substances, but such samples may be commonly available at the drug black market, we have developed a method for their identification with X-ray powder diffraction (XRPD), which also enabled us to distinguish between different polymorphs/solvates of methoxphenidine that were crystallized in the laboratory. XRPD offers additional data about samples, which may not be discovered by routine techniques, and in some cases, they may help to find out essential information.

## 1. Introduction

Chemical modifications of illicit drugs result in novel compounds commonly known as new psychoactive substances (NPSs) or designer drugs that circumvent legislation. When the pharmacophore of a thus-modified compound is preserved, it usually mimics the biological effects of the parental drug, but with unexplored pharmacological effects and toxicity. By the end of 2020, the European Monitoring Centre for Drugs and Drug Addiction (EMCDDA) had identified more than 830 NPSs [1]. The ever-increasing number of NPSs on the black market makes it clear that we are only at the very tip of a substance-abuse iceberg.

EMCDDA divides NPSs into several groups, one of which includes dissociative anesthetics. Although dissociative anesthetics represent a relatively small group of NPSs, they are often abused for their wide range of mind-altering effects. Unfortunately, in some instances, such abuse has resulted in human deaths [2]. Despite this, the recently discovered dissociative anesthetic potential for the treatment of depression has brought this group of substances to the forefront of medical interest [3,4,5,6,7,8]. However, the long-term use of ketamine, the most abused dissociative anesthetic, has been directly linked to various severe adverse effects, including bladder inflammation [9,10]. Therefore, wider utilization of dissociative anesthetics in clinical practice has been limited, so far. However, a deeper understanding of the toxicological profile and behavior of these compounds in biological systems may help in the development of a structure–activity relationship (SAR) model and, thereby in the design of new pharmaceuticals with improved performance and suppressed side effects.

**Figure 1:**
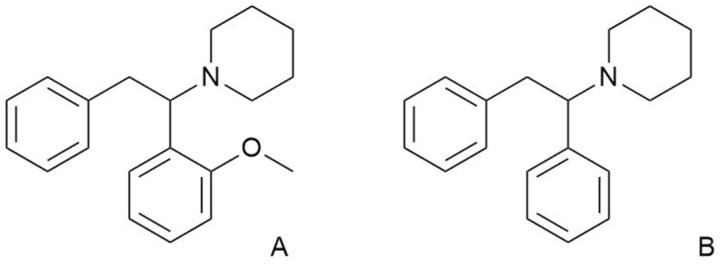
Chemical structures of (**A**) methoxphenidine, (**B**) diphenidine.

Methoxphenidine (MXP; 1-[1-(2-methoxyphenyl)-2-phenylethyl]piperidine), a structural analogue of diphenidine (Figure 1), is an NPS belonging to the group of dissociative anesthetics. It is an *N*-methyl-D-aspartate receptor antagonist [11,12] with a mild affinity to the norepinephrine transporter [13], a weaker one to the dopamine transporter [13], and almost no affinity to the serotonin transporter [11]. It was originally patented in 1989 as a substance with potential neuroprotective effects and began to be abused approximately two decades later, probably in reaction to the regulation of other dissociative anesthetics [11,14].

Reports of MXP being sold on the black market emerged from the United Kingdom in 2013 and were quickly followed by news of its seizure in several countries of the European Union (EU) [15,16]. MXP intoxications have been reported in several EU countries [16,17,18,19], and it is known as a cause of at least three deaths [20]. Users usually take the MXP orally; however, other administration options such as sublingual or intranasal were also described [21]. It is worth noting that MXP doses very often exceed 100 mg [21]. These users describe the effects of MXP as the clear-headed state in which they have difficulty focus on tasks, relaxation with a decrease in anxiety, dreamy state, altered cognitive effects, warmth, euphoria, and sensations of the senses, blurred vision, internal hallucinations, time and space distortion, difficulty moving, pain relief, a dissociative state, feeling as if they transformed into pure energy and seeing pictures in the universe [21,22]. Within 24 h after the effect, an afterglow may occur which may be as desirable as the effect itself [22]. However, MXP abuse has been associated with rhabdomyolysis and acute kidney injury [23]. MXP may also be especially dangerous in combination with other substances. For instance, in a combination with depressants including alcohol or GHB/GBL it may lead to respiratory depression, loss of consciousness up to death [22]. A combination with stimulants may induce anxiety, mania, delusions, or psychotic episode [22]. MXP in combination with other dissociatives may lead to delusions, sedation, amnesia, nausea, and even respiratory depression [22]. Moreover, likewise, with other dissociative anesthetics [24,25], methoxphenidine may also be addictive [22,26,27] and may produce various symptoms related to schizophrenia [26]. Yet, even though it has already been available on the black market for at least half a decade, there remains a lack of information on its pharmacological and physico–chemical properties.

In this study, we solved the single-crystal structure of an MXP sample obtained from an online drug vendor. Interestingly, we found that inorganic impurities, which may have unexpected effects on an abuser’s health, were incorporated into this crystal structure. Consequently, we synthesized a standard of this compound and compared the in vitro toxicological properties of the standard with those of the street sample.

## 2. Materials and Methods

### 2.1. Material and Chemicals

Sample I. was obtained from the now-defunct website Astro-lab.com (10 March 2017). Sample II. was obtained within a collaboration with the Czech border police. A standard of MXP for comparison was prepared using the McLaughlin procedure [15], which was slightly modified to obtain MXP without any inorganic impurities (see Section 2.4). Chemicals used for the synthesis were obtained from commercial sources (Sigma-Aldrich, St. Louis, MO, USA). Solvents were acquired from commercial sources and were used after distillation. Solvents denoted as dry were dried before use by molecular sieves. Other commercial reagents were used without further purification. The structure of the product was confirmed by ^1^H and ^13^C-NMR spectroscopy (400-MR DDR2 spectrometer, Agilent, Santa Clara, CA, USA), deuterated methanol was used as a solvent and the residual signals of the solvent served as an internal standard. The diethyl ether solution of hydrochloride was prepared from commercial diethyl ether, commercial concentrated hydrochloric acid, and calcium chloride as drying agents.

### 2.2. HPLC Analysis

Gemini 5µ C18 column (250 × 4.6 mm, Phenomenex, Torrance, CA, USA) with a guard column was used for the separation street MXP sample. The mobile phase consisted of A: 5% MeOH in water containing 1% TFA (*v*:*v*:*v*) and B:1% TFA (*v*:*v*) in MeOH. Gradient elution: 30% D (0 min), increasing linearly to 100% D (20 min). The gradient mobile phases were degassed continuously by sparking with helium at a rate of 40 mL·min^−1^. The HPLC system (Waters, Milford, MA, USA) consisted of a pump equipped with a 600E system controller, autosampler 717, and dual UV detector 2487. The data were processed with Empower 2 software (Waters Corp., Milford, MA, USA). UV detection was carried out at 279 nm [28].

### 2.3. Mass Spectrometry

Mass spectrometric experiments were performed using a commercial 9.4T APEX-Ultra FTMS instrument (Bruker Daltonics, Billerica, MA, USA) equipped with an electrospray ionization/matrix-assisted laser desorption/ionization (ESI/MALDI) ion source. The analysis was performed using ESI and the spectra were acquired in positive ion mode. The detailed settings of the analysis can be found in a recent publication [29].

### 2.4. Synthesis of Methoxphenidine

The reaction was performed under an inert atmosphere. Benzyl bromide (1.6 mL, 0.3 eq) was added into a suspension of 8 g (2.8 eq) zinc in dry acetonitrile (70 mL) followed by dropwise addition of CF_3_COOH (1.25 mL, 0.3 eq) in 5 mL of acetonitrile at room temperate (RT). The suspension was stirred for 15 min. which was followed by simultaneous addition of benzyl bromide (12 mL, 2.3 eq), dry piperidine (4.8 mL, 1.1 eq), and dissolved 2-methoxy benzaldehyde (6 g, 1.0 eq) each in 5 mL of acetonitrile while keeping the mixture at RT. The reaction was monitored with the aid of thin-layer chromatography (TLC, Silica gel 60 F254). After 2 h of vigorous stirring, the reaction was quenched by the addition of 500 mL of 2 M KOH solution, extracted by dichloromethane (3 × 150 mL), and the combined organic phases were rinsed with saline. The organic phase was dried over MgSO_4_, filtered, and evaporated to dryness using a rotavap and an oil pump. The obtained 15.3 g of dark oil was diluted with 100 mL of Et_2_O and extracted to 3 M HCl (3 × 80 mL). Activated carbon was added into the combined water phases and the suspension was stirred for 30 min. Then, the suspension was filtered through celite and basified by the addition of saturated NaOH solution, which was added until the pH was basic. The water phase was extracted by Et_2_O (3 × 120 mL), dried over MgSO_4_, filtered, and then evaporated to dryness. Yellowish oil was then purified by flash chromatography (hexane/AcOEt/Et3N: 6/1/0.07) to obtain 5.9 g of colorless liquid (yield 45%). To obtain MXP hydrochloride, 3.3 g of MXP was diluted by 15 mL of Et_2_O and then, hydrochloride in Et_2_O was added dropwise (8 mL). The obtained suspension was evaporated to dryness and the product was crystallized from acetone yielding 1.2 g of colorless crystalline material. For XRPD measurement, the standard of MXP was also recrystallized from chloroform or a combination of acetonitrile/diethyl ether yielding white crystalline material. The NMR spectra corresponded to ones reported in ref. [15].

### 2.5. Single-Crystal X-ray Diffraction

The structural data were collected using Bruker D8 VENTURE system equipped with a Photon 100 CMOS detector, a multilayer monochromator, and a CuKα Incoatec microfocus sealed tube (*λ* = 1.54178 Å) using combined *φ* and *ω* scans at 180 K. 

The structure of sample I. was in a monoclinic system, *C* 2/*c* space group with lattice parameters *a* = 24.6376(18) Å, *b* = 12.7072(10) Å, *c* = 17.5197(14) Å, *α* = 90°, *β* = 132.211(4)°, *γ* = 90°, *Z* = 4, *V* = 4062.6(6) Å^3^. The data reduction and absorption correction were performed with Apex3 software. The structure was solved by charge flipping methods using Superflip software and refined by full-matrix least squares on *F* squared value using Crystals software to final values of *R* = 0.1906 and *wR* = 0.2396 using 3598 independent reflections (*Θ_max_* = 66.567°), 242 parameters and 24 restraint. MCE software was used for the visualization of residual electron density maps. According to common practice, the hydrogen atoms attached to carbon atoms were placed geometrically with *U*_iso_(H) in range 1.2–1.5 *U*_eq_ of parent atom (C). The inorganic anions lied on the two-fold axes. Atoms of bromide and chlorine were at similar positions with partial occupation in the average cell, so we constrained the position and thermal ellipsoids of chlorine atoms to corresponding bromine atoms. The structure was deposited into the Cambridge Structural Database under the number CCDC 2075030.

A 10 mg of sample I. was recrystallized from a 0.5 mL MeOH for two weeks (storage in an NMR tube in the fridge) and measured. It was in a monoclinic system, *P* 2_1_/*c* space group with lattice parameters *a* = 16.3779(13) Å, *b* = 24.820(3) Å, *c* = 11.4760(18) Å, *α* = 90°, *β* = 109.610(5)°, *γ* = 90°, *Z* = 4, *V* = 4394.4(9) Å^3^, *D_c_* = 1.301 g·cm^−3^ (without solvent), *μ*(Cu-Kα) = 3.954 mm^−1^. The data reduction and absorption correction were performed with Apex3 software. The structure was solved by charge flipping methods using Superflip software and refined by full-matrix least squares on *F* squared value using Crystals software to final values of *R* = 0.0320 and *wR* = 0.0762 using 8321 independent reflections (*Θ_max_* = 70.058°), 447 parameters and one restraint. MCE software was used for the visualization of residual electron density maps. According to common practice, the hydrogen atoms attached to carbon atoms were placed geometrically with *U*_iso_(H) in range 1.2–1.5 *U*_eq_ of parent atom (C). Atoms of bromide and chlorine are at similar positions with partial occupation in the average cell, so we constrained the position and thermal ellipsoids of chlorine atoms to corresponding bromine atoms. The contribution of the solvent molecules was subtracted from the observed intensities using the SQUEEZE routine in software Platon. The structure was deposited into the Cambridge Structural Database under the number CCDC 1913081.

The methoxphenidine chloroform solvate (the standard recrystallized from chloroform) structure was in a monoclinic system, *P* 2_1_/*n* space group with lattice parameters *a* = 9.6408(3) Å, *b* = 24.2751(8) Å, *c* = 9.9665(3) Å, *α* = 90°, *β* = 102.8238(13)°, *γ* = 90°, *Z* = 4, *V* = 2274.30(12) Å^3^, *D_c_* = 1.318 g·cm^−3^, *μ*(Cu-Kα) = 4.809 mm^−1^. The frames were integrated with the Bruker SAINT software package, the absorption effects were corrected using the Multi-Scan method (SADABS). The ratio of minimum to the maximum apparent transmission was 0.488. The structure was solved by charge flipping methods using Superflip software and refined by full-matrix least squares on *F* squared value using Crystals software to final values *R* = 0.0499 and *wR* = 0.1045 using 3777 independent reflections (*Θ_max_* = 68.237°), 249 parameters and one restraint. All H-atoms were located in a difference map but repositioned geometrically, then they were initially refined with soft restraints on the bond lengths and angles to regularize their geometry (C-H in the range of 0.93–0.98 Å, N-H set to 0.86 Å) and Uiso(H) (in the range of 1.2–1.5 times U_eq_ of the parent atom), after which the positions of carbon-bound hydrogen atoms were refined with riding constraints. The positional parameters and Uiso of the N-bound hydrogen atom were refined. The structure was deposited into the Cambridge Structural Database under the number CCDC 2078796.

### 2.6. X-ray Powder Diffraction

A standard-oriented silicone pod was used for the X-ray powder diffraction measurement. The crystals were crushed on the pod by a glass microscope slide and narrowed to form a flat surface. The data were collected using a Bruker D2 Phaser powder diffractometer. The measurements were performed at room temperature with Bragg–Brentano geometry using Co-Kα (λ = 1.79 Å) radiation with a step of 0.02° 2*θ* and 0.8 s measurement time for each step. LYNXEYE XE detector was used for signal collection. The data were scanned over the angular range of 5° to 85° 2*θ*. The diffraction patterns were processed by High Score Plus 3.0e (PANalytical, Almelo, The Netherlands).

### 2.7. Identification of the Impurity in MXP

Carbon content was determined by the Vario TOC Cube machine with an NDIR detector. Calibration standard ASTASOL was used for quantification within the calibration range.

Chloride content was determined by Ion chromatography machine IC 930, column Metrostep A Sup 7250 × 4 mm, column temperature of 45 °C with the conductometric detector. A calibration curve of analytical standards for chlorides was used for chloride quantification.

Sample for zinc determination was prepared by taking a 10 mL aliquot of the sample and acidified by 0.1 mL of 65% HNO_3_ and determined by inductively coupled plasma mass spectrometry (ICP-MS; Agilent 7900ISIS3, plasma gas flow of 12 L·min^−1^, auxiliary argon gas flow of 1.2 L·min^−1^, the input of 1.5 kW) A calibration curve of the analytical standard of zinc was used for zinc quantification.

A sample for the determination of bromides was prepared by taking a 10 mL aliquot of the sample and preserved with 0.1 mL of 25% tetramethylammonium hydroxide (TMAH) solution. The measurement was performed by ICP-MS (Agilent 7900ISIS3, plasma gas flow of 12 L·min^−1^, auxiliary argon gas flow of 1.2 L·min^−1^, input of 1.5 kW). A calibration curve of the analytical bromine standard was used for bromine quantification.

### 2.8. Cell Line Cultivation

The following cell lines were used in this study: Hep G2, HMC-3, 5637, SH-SY5Y, A549, MRC-5, L929, RAW 264.7, and NIH 3T3. In total, there were six human cell lines used, which were derived from hepatocellular carcinoma (Hep G2), microglia (HMC-3), urinary bladder grade II carcinoma (5637), neuroblastoma (SH-SY5Y), lung carcinoma (A549), and primary noncancerous lung cells (MRC-5). Aside from these, four mouse cell lines were also used: fibroblasts (L929 and NIH 3T3), macrophages (RAW 264.7), and myoblasts (C2C12). The abovementioned cell lines were chosen so that the most often affected tissues by NPS are represented, such as liver, kidney, urinary bladder, lungs as well as cells derived from nervous tissues. Moreover, primary noncancerous cells, MRC-5, also were utilized. The cell lines were supplied from American Tissue Culture Collection except for L929, MRC-5, and RAW 264.7, which were purchased from Sigma-Aldrich. All the cell lines were maintained as recommended by the supplier, i.e., at the exponential phase of growth in the recommended cultivation media supplemented with 10% fetal bovine serum (certified; Thermo Fisher Scientific, Waltham, MA, USA). The cells were cultivated at sterile conditions in an incubator at 37 °C, with 5% CO_2_, and 95% humidity. The cells were routinely morphologically examined and time to time tested for mycoplasma.

### 2.9. MXP Cytotoxicity Evaluation

In vitro cytotoxicity of both street and standard MXP was evaluated by a viability WST-1 assay (Sigma-Aldrich, St. Louis, MO, USA). For each cell line (listed in Section 2.8) measured, the amount of 5000 to 10,000 cells (depending on the individual cell line) per well was seeded into single wells of 96-well in 100 µL of complete cell cultivation media. Then, the cells were cultured for 16 h at 37 °C, 5% CO_2_, and 95% humidity, after which they were treated with 100 µL of fresh cell cultivation media, which was added to the 100 µL of media already contained in the wells, containing the evaluated compounds at 0–1 mM concentrations. Then, the cell viability was determined 72 h after treatment with the following procedure: the culture medium was discarded and changed for 100 µL of fresh phenol red-free Dulbecco’s Modified Eagle Medium (DMEM) with the addition of 10 µL of WST-1 reagent. After that, the cells were incubated for two more hours. Then, the absorbance of raised formazan was spectrophotometrically measured at 450 nm (ref. at 630 nm) using a UV-Vis spectrometer (Bio-Rad Laboratories, Hercules, CA, USA). All samples were tested in quadruplicates. Untreated cells and cells treated with a vehicle served as controls. From the dose-response curve, the IC_50_ values were calculated using AAT Bioquest.

### 2.10. Sample Preparation for Fluorescence Microscopy

The amount of 1 × 10^5^ of 5637 and SH-SY5Y cells was seeded into 35 mm glass-bottom dishes (#1.5) for live-cell imaging (MatTek, Ashland, MA, USA) and kept to adhere for 16 h. After that, the cells were gently washed twice with prewarmed (37 °C) phosphate-buffered saline (PBS; pH 7.4) which was changed for DMEM supplemented with street and standard MXP sample at the final concentration of 150 and 300 µM for 5637 cells, and 50 and 100 µM for SH-SY5Y cells. The incubation with the evaluated compounds proceeded for 24 h; untreated cells served as controls. After the incubation period, the cells were gently washed with PBS, and then the cells were fixed with a 4% solution of methanol-free formaldehyde (Thermo Fisher, Waltham, MA, USA) in PBS for 20 min in the dark at laboratory temperature. Then the fixative was discarded and the cells were washed twice with PBS and stained with a solution of phalloidin-Atto 488 (Atto-tec, Siegen, Germany) with 4’,6-diamidino-2-phenylindole (DAPI, Sigma-Aldrich, St. Louis, MO, USA) for 15 min. After that, the staining solution was discarded, the cells were washed twice with PBS and subjected to microscopy analysis.

### 2.11. Fluorescence Microscopy

The impact of both street and standard MXP samples on 5637 and SH-SY5Y cell morphology was examined by wide-field fluorescence microscopy using an Olympus IX-81 microscope (Olympus, Tokyo, Japan). The images were acquired by EM-CCD camera C9100-02 (Hamamatsu, Nakaku, Japan) using xCellence software, 60× oil immersion objective with the NA of 1.4 and high-stability 150 W xenon burner. The images were background-corrected and deconvolved by 2D deconvolution with the no-neighbor algorithm.

### 2.12. Apoptosis/Necrosis Assay

The impact of both street and standard MXP samples on cell death type induced in 5637, SH-SY5Y, and MRC-5 cells was studied by using an apoptosis/necrosis kit (Abcam, Cambridge, UK) following the manufacturer protocol. Briefly, the cells were seeded as in the cytotoxicity evaluation described in Section 2.9, but instead of WST-1, the apoptosis/necrosis kit was added, i.e., the cells were washed with 100 µL of assay buffer and, then, 200 µL of assay buffer with 2 µL of apopxin green indicator (100×; labeling apoptotic cells) and 1 µL of 7-AAD (200×; for visualization of necrotic cells as well as late apoptotic cells) were added to the cells per one well for 30 min. After that, the cells were washed with PBS and fixed with 2% formaldehyde solution in PBS and subjected to fluorescence microscopy to capture apoptotic and necrotic cells, the samples were prepared in three replicates, from each 6–10 region of interest were monitored and the results were evaluated by Image J. Untreated cells served as a control.

## 3. Results and Discussion

**Figure 2:**
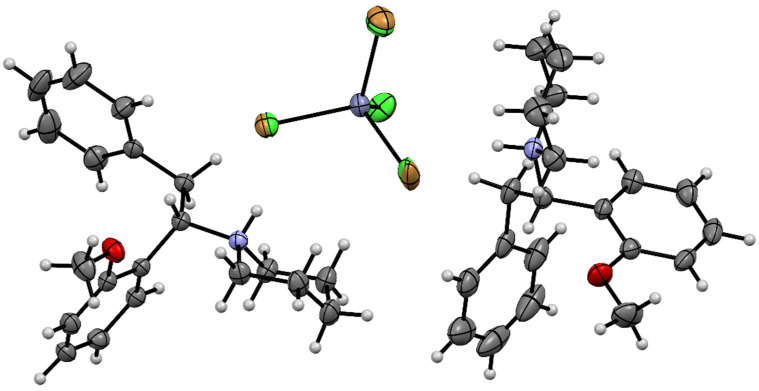
Single-crystal structure of sample I. which was recrystallized from methanol.

The now-defunct website Astro-lab.com sold us 5 g of a sample, which was labeled as MXP. The sample was a solid white material, which underwent a standard identification procedure in our forensic laboratory. During the MS and ^1^H and ^13^C NMR analyses, we confirmed that the sample contained MXP. By the HPLC-UV analysis, we determined the sample purity was 95%. However, the sample was poorly soluble in either methanol or water, which was not common for similar substances. As the sample was solid crystalline powder, a measurement by X-ray diffraction could further confirm the presence of MXP in the structure and possibly help to explain the nonstandard properties of the sample. Despite the sample being polycrystalline, a single crystal was found within the sample and measured by single-crystal X-ray diffraction. MXP was indeed present in the crystal; however, the sample did not contain usual inorganic counter ions such as chlorine, bromine, or sulfate. The data suggested that the counter ion was a zinc complex containing both chlorine and bromine. As the quality of the obtained single crystals was not satisfactory to obtain a sufficient dataset from X-ray diffraction, the sample was recrystallized from methanol and the obtained single crystal was measured (see Figure 2). These additionally obtained data suggested that the counter ion in the single-crystal contained ZnBr_1_Cl_3_^2−^ or ZnBr_2_Cl_2_^2−^ in 60 and 40% of cases, respectively (halogens were altering in the crystal). To confirm the inorganic counter ion by other methods, the sample was measured by energy-dispersive X-ray spectroscopy (EDS), X-ray fluorescence (XRF), and other methods (see Section 2.7). From the obtained data, we suggest that the inorganic anion was predominantly ZnBr_2_Cl_2_^2−^.

**Figure 3:**
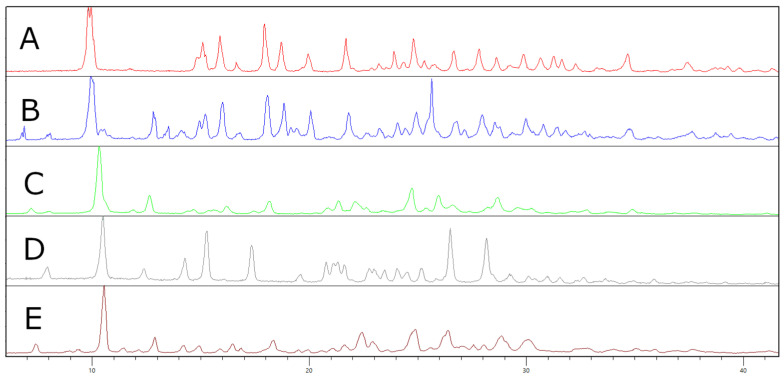
Diffractograms of (**A**) a commercial sample of methoxphenidine (MXP); (**B**) a sample of MXP obtained from Czech border police; (**C**) MXP hydrochloride recrystallized from acetone; (**D**) MXP hydrochloride recrystallized from chloroform; (**E**) MXP hydrochloride recrystallized from acetonitrile/diethyl ether.

Since the activity and toxicity differences between samples with such impurities, possibly causing severe health issues, may exist, the ability to differentiate between these samples may be of concern for forensic laboratories. For this reason, we employed X-ray powder diffraction and measured the obtained commercial sample plus another sample gained from the Czech law-enforcement agencies [30]. Based on the similarity of the diffraction patterns, we suggest that these samples may contain the same inorganic impurity as sample I. The presence of the zinc counter ion in these samples was later confirmed by XRF. To demonstrate the differences in the diffraction patterns between MXP samples containing the zinc complex and MXP hydrochloride, we also measured the XRPD of an in-house prepared standard, which was crystallized from acetone, chloroform, and a combination of acetonitrile/diethyl ether (see Figure 3). The observed differences in diffractograms C, D, and E were caused by yielding different either polymorphs or solvates via the recrystallization. Furthermore, as the recrystallization using chloroform yielded a single crystal, we also solved the structure using single-crystal X-ray diffraction.

As the sample containing this inorganic counter ion was available on the market, we decided to compare the sample toxicity with an in-house prepared standard of MXP hydrochloride (see Section 2.4).

The in vitro cytotoxic profiles of the street MXP and its standard were assessed using a viability assay of WST-1 after 72 h of cell treatment. Cell lines of various origins were selected for this cytotoxicity evaluation to assess hepatic, urinary, renal, cardiac, lung, and neuronal toxicity, for which the following human cell lines were used Hep G2, 5637, C2C12, A549, MRC-5, SH-SY5Y, and HMC-3, respectively. Moreover, three noncancerous cell lines, RAW 264.7, L929, and NIH 3T3, of mouse origin mimicking healthy tissues were also used. The IC_50_ values of street MXP and its standard were determined after 72-h treatment of cells of the individual cell lines, see Table 1.

The in vitro cytotoxicity of both street and standard MXP was concentration dependent and the IC_50_ values were in micromolar concentrations. Surprisingly, we detected substantial differences in the cytotoxicity of both compounds, with the biggest contrast detected in human cells derived from the urinary bladder (5637), neuroblastoma (SH-SY5Y), lungs (A549), and also in human primary noncancerous fibroblasts (MRC-5) and mouse macrophages (RAW 264.7). In these cell lines, the street MXP exhibited almost double toxicity compared to the standard MXP, which was very likely caused by the inorganic impurities contained in the street sample of MXP. Based on the fact that MXP is a weakly potent NPS so the abused dose must be much higher to achieve the desired effect, such undesired inorganic impurities found by us in the street MXP can be a big health hazard to drug abusers.

**Figure 4:**
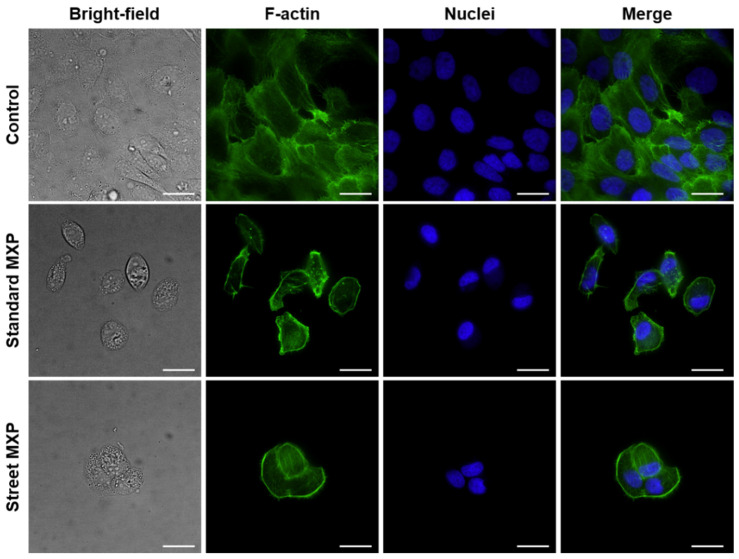
Fluorescence microscopy images of human cells derived from urinary bladder carcinoma (5637) treated with a street sample of methoxphenidine (MXP) containing inorganic impurities and its synthetic standard. The 5637 cells were treated with a 150 µM concentration of both MXP for 24 h. Individual columns from left: bright-field images, cell cytoskeleton labeled with phalloidin-Atto 488, cell nuclei stained with DAPI, and merges of the fluorescent images. The scale bars correspond to 20 µm; 60× objective was used.

**Figure 5:**
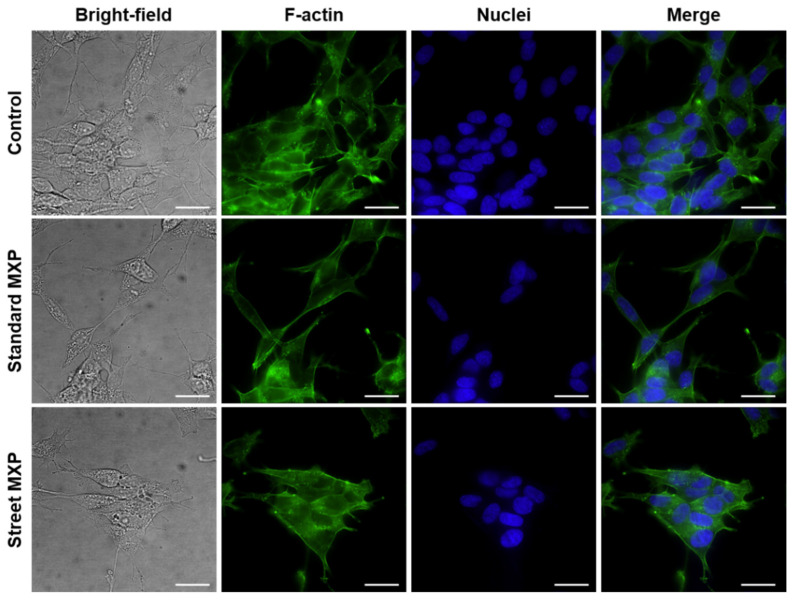
Fluorescence microscopy images of human cells derived from neuroblastoma (SH-SY5Y) treated with a street sample of methoxphenidine (MXP) containing inorganic impurities and its synthetic standard. The SH-SY5Y cells were treated with a 100 µM concentration of both MXP for 24 h. Individual columns from left: bright-field images, cell cytoskeleton labeled with phalloidin-Atto 488, cell nuclei stained with DAPI, and merges of the fluorescent images. The scale bars correspond to 20 µm, 60× objective was used.

The 5637 and SH-SY5Y cell lines, in which was detected the highest toxicity together with the biggest differences between the street and standard MXP, were further subjected to microscopy analysis, in which the cell cytoskeleton and cell nuclei were stained. It is apparent from the fluorescence microscopy images in Figure 4 and Figure 5 not only that there were fewer cells in the samples treated with both variants of MXP, but also the cell morphology was significantly affected after the MXP treatment. The 5637 cells treated with 150-µM MXP were roundish and of pre-apoptotic morphology (bright-field images) and the actin cytoskeleton was more tightly packed with contents of F-actin granules. SH-SY5Y cells appeared bigger and with longer protrusions after both street and standard MXP treatment. Similarly, as in the case of 5637, there were much fewer cells than on untreated control. In addition, in bright-field images of SH-SY5Y cells treated with MXP, the granularity of the cytoplasm was apparent which indicates the unhealthy state of the cells.

**Figure 6:**
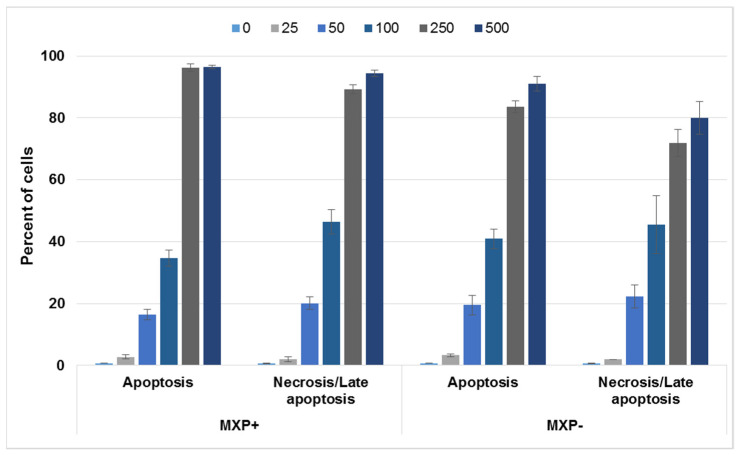
Cell death induction in human cells derived from urinary bladder carcinoma (5637) treated with a street sample of methoxphenidine (MXP) containing inorganic impurities (MXP+) and its synthetic standard (MPX-). The 5637 cells were treated with a 0–500 µM concentration of both MXP for 72 h, after which fluorescence detection of apoptotic and necrotic/late apoptotic cells was determined. The error bar is standard deviation from three samples.

**Figure 7:**
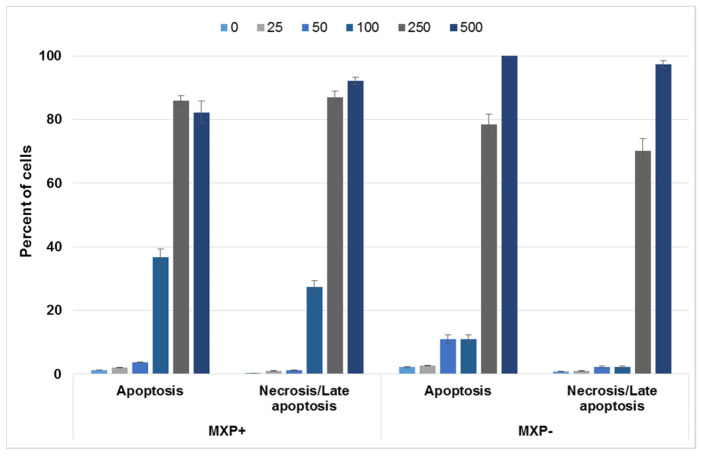
Cell death induction in human cells derived from neuroblastoma (SH-SY5Y) treated with a street sample of methoxphenidine (MXP) containing inorganic impurities (MXP+) and its synthetic standard (MPX-). The SH-SY5Y cells were treated with a 0–500 µM concentration of both MXP for 72 h, after which fluorescence detection of apoptotic and necrotic/late apoptotic cells was determined. The error bar is standard deviation from three samples.

Further, based on the observed pre-apoptotic morphology of cells treated with MXP, we aimed to examine the type of cell death induced by this NPS. For this experiment, again 5637 and SH-SY5Y cell lines were chosen and treated either with MXP containing inorganic impurities or its synthetic standard at 0–500 µM concentrations for 72 h, after which the cells were stained with an apoptosis/necrosis fluorescent kit. The proportion of apoptotic vs. necrotic 5637 and SH-SY5Y cells after this treatment is given in Figure 6 and Figure 7, respectively. As it is apparent from Figure 6, there was a slightly bigger proportion of 5637 cell in the apoptotic and necrotic state upon treatment with MXP with inorganic impurities than when treated with the synthetic standard. As for SH-SY5Y cells, there was significantly higher proportion of apoptotic and necrotic cells at 100 and 250 µM treatment with MXP containing inorganic impurities than compared to the synthetic standard. The reason why there were such a big proportion of cells in both apoptosis and necrosis at the two highest concentrations can be explained by the fact that the 7-AAD dye for necrotic cells also labels late stages of apoptosis. Next, cell confluency was also monitored at the same conditions and concentrations of MXP used for treatment after 72 h of incubation, the data for both cell lines and both compounds are shown in Supplementary Materials in Figure S1, there was an inverse correlation of the cell number with the increasing concentration of the MXP.

The inorganic impurity we identified in the MXP street sample was somewhat surprising as it was the counter ion of the MXP salt and to the best of our knowledge, we are the first to detect such a zinc-based inorganic impurity in NPS. Psychoactive substances are usually diluted with organic substances that either alter the effect or potency of the product (so-called adulterants) or by dilutants, which have the main task of increasing the seller’s profit [31,32,33,34,35,36,37,38]. In some cases, intermediates or impurities from the synthesis [39,40] or isolation [41] of respective psychoactive substances may be also present in the samples. In contrast, inorganic impurities (such as laundry detergent or boric acid in cocaine [42]), which can also be classified as dilutants, are slightly less common in drugs.

The inorganic impurities, however, can be more difficult to detect, because if either MS, LC/MS, or LC-DAD are used for the routine analysis without other methods, the impurities may remain hidden. For these purposes, it is therefore essential to also implement other instrumental techniques that can detect these inorganic impurities [43]. X-ray powder diffraction is particularly suitable for this purpose [44,45,46,47]. Not only can it identify the inorganic impurity in the drug sample, but it can also distinguish whether such an inorganic impurity is part of a crystal lattice (as shown in our article) or whether the sample is solely a mixture of substances. Taken together with the ability of XRPD to distinguish between the polymorphs, XRPD may provide several fruitful data from a single analysis [48]. To exploit the entire potential of the X-ray powder diffraction without the need of using a standard in the measurement, it is necessary to create a database of psychoactive substances and common diluents/adulterants [44,49].

Since we found the impurity in both samples from the black market, we tried to discover in anecdotal reports and forums whether users did not complain about the rather low purity of this NPS. We discovered that users mentioned the relatively slow onset of the orally ingested alleged MXP hydrochloride and an increase in blood pressure [50,51]. They noted that if they formed a free base and made citrate instead, the onset of the effect was significantly faster and did not alter the blood pressure that much [50]. Such behavior of the MXP may be explained by this zincate ion in its structure and these users in this way could unknowingly observe a difference between the behavior of this salt compared to the expected properties of the hydrochloride.

## 4. Conclusions

We have discovered and structurally described a novel psychoactive drug, MXP, traded on the internet market, which was contaminated with unusual inorganic impurities. It can be described as a salt of MXP with a mixed bromo- and chloro-zincate complex. As expected, the in vitro toxicity screening of the drug in a series of cell lines showed dramatically higher toxicity compared to our synthesized standard. Considering the weak potency of this NPS demanding higher doses when abused, it can cause severe harm to the consumers. As this kind of contamination could be virtually invisible for routine spectroscopy methods, XRPD should be preferred as the first-choice method for such detection. Furthermore, XRPD can easily distinguish a variety of drug forms such as solvates and polymorphs and, thus, it is able to provide more information on the sample history. Combinations of forms and impurities provide fingerprints that may be unique for the particular batch/source.

## Figures and Tables

**Table 1:** Evaluation of in vitro cytotoxicity of street methoxphenidine sample and its standard after 72-h cell treatment. IC_50_–concentration of a compound required to achieve 50% reduction of cell viability; SD—standard deviation (from three replicates).

Cell Line	Street MXP	MXP Standard
	IC5_0_ ± SD [µM]	
5637	59.74 ± 1.89	101.73 ± 2.81
SH-SY5Y	49.74 ± 2.70	97.94 ± 3.64
RAW 264.7	86.35 ± 3.10	137.65 ± 5.43
MRC-5	87.52 ± 3.41	123.85 ± 2.03
A549	154.45 ± 5.33	180.31 ± 1.13
HMC-3	117.93 ± 6.71	132.80 ± 7.57
Hep G2	71.12 ± 1.01	79.18 ± 2.98
L929	144.33 ± 4.48	155.72 ± 1.91
NIH 3T3	86.36 ± 7.84	95.12 ± 8.1
C2C12	99.97 ± 5.38	105.23 ± 3.19

## Data Availability

Not applicable.

## Supplementary Materials

The following are available online at https://www.mdpi.com/article/10.3390/ijms23042083/s1.

## Abbreviations

5637	Human cells from urinary bladder grade II carcinoma
A549	Human cells from lung carcinoma
C2C12	Mouse myoblasts
DAPI	4′,6-diamidino-2-phenylindole
DMEM	Dulbecco’s Modified Eagle Medium
EDS	Energy-dispersive X-ray spectroscopy
EMCDDA	European Monitoring Centre for Drugs and Drug Addiction
ESI	Electrospray ionization
Hep G2	Human cells from hepatocellular carcinoma
HMC-3	Human microglial cells
ICP-MS	Inductively coupled plasma mass spectrometry
L929	Mouse fibroblasts
MALDI	Matrix-assisted laser desorption/ionization
MRC-5	Human primary fibroblasts
MXP	Methoxphenidine
NIH 3T3	Mouse fibroblasts
NPSs	Psychoactive substances
RAW 264.7	Mouse macrophages
RT	Room temperate
SH-SY5Y	Human cells from neuroblastoma
TLC	Thin-layer chromatography
XRF	X-ray fluorescence
XRPD	X-ray powder diffraction
