# Supplementary material for: Intriguing Cytotoxicity of the Street Dissociative Anesthetic Methoxphenidine: Unexpected Impurities Spotted
Source: Int J Mol Sci. 2022 Feb 14;23(4):2083. doi: 10.3390/ijms23042083 (PMC8879332; doi:10.3390/ijms23042083)
Supplement: Supplementary file 1 [file ijms-23-02083-s001.zip › ijms-1463671-supplementary.pdf]

International Journal of *Molecular Sciences*

MDPI

Article

Supplementary information

Intriguing cytotoxicity of the street dissociative anesthetic methoxphenidine: Unexpected impurities spotted

Bronislav Jurásek [1,2], Silvie Rimpelová [3,*], Martin Babor [4], Jan Čejka [4], Vilém Bartůněk [5] and Martin Kuchař [1,2,*]

[1] Forensic Laboratory of Biologically Active Substances, Department of Chemistry of Natural Compounds University of Chemistry and Technology Prague, Technická 5, 166 28, Prague 6, Czech Republic; bronislav.jurasek@vscht.cz; martin.kuchar@vscht.cz
[2] Department of Experimental Neurobiology, National Institute of Mental Health, Topolová 748, 250 67 Klecany, Czech Republic
[3] Department of Biochemistry and Microbiology, University of Chemistry and Technology Prague, Technická 5, 166 28, Prague 6, Czech Republic; silvie.rimpelova@vscht.cz
[4] Department of Solid State Chemistry, University of Chemistry and Technology Prague, Technická 5, 166 28 Prague 6, Czech Republic; martin.babor@vscht.cz; jan.cejka@vscht.cz
[5] Department of Inorganic Chemistry, University of Chemistry and Technology Prague, Technická 5, 166 28 Prague 6, Czech Republic; vilem.bartunek@vscht.cz
* Correspondence: martin.kuchar@vscht.cz (M.K.); silvie.rimpelova@vscht.cz (S.R.)

Citation: Jurásek, B.; Rimpelová, S.; Babor, M.; Čejka, J.; Bartůněk, V.; Kuchař, M. Intriguing Cytotoxicity of the Street Dissociative Anesthetic Methoxphenidine: Unexpected Impurities Spotted. *Int. J. Mol. Sci.* **2022**, *23*, 2083. https://doi.org/10.3390/ijms23042083

Academic Editor: Rosaria Acquaviva

Received: 29 October 2021
Accepted: 11 February 2022
Published: 14 February 2022

Publisher's Note: MDPI stays neutral with regard to jurisdictional claims in published maps and institutional affiliations.

Copyright: © 2022 by the author. Licensee MDPI, Basel, Switzerland. This article is an open access article distributed under the terms and conditions of the Creative Commons Attribution (CC BY) license (https://creativecommons.org/licenses/by/4.0/).

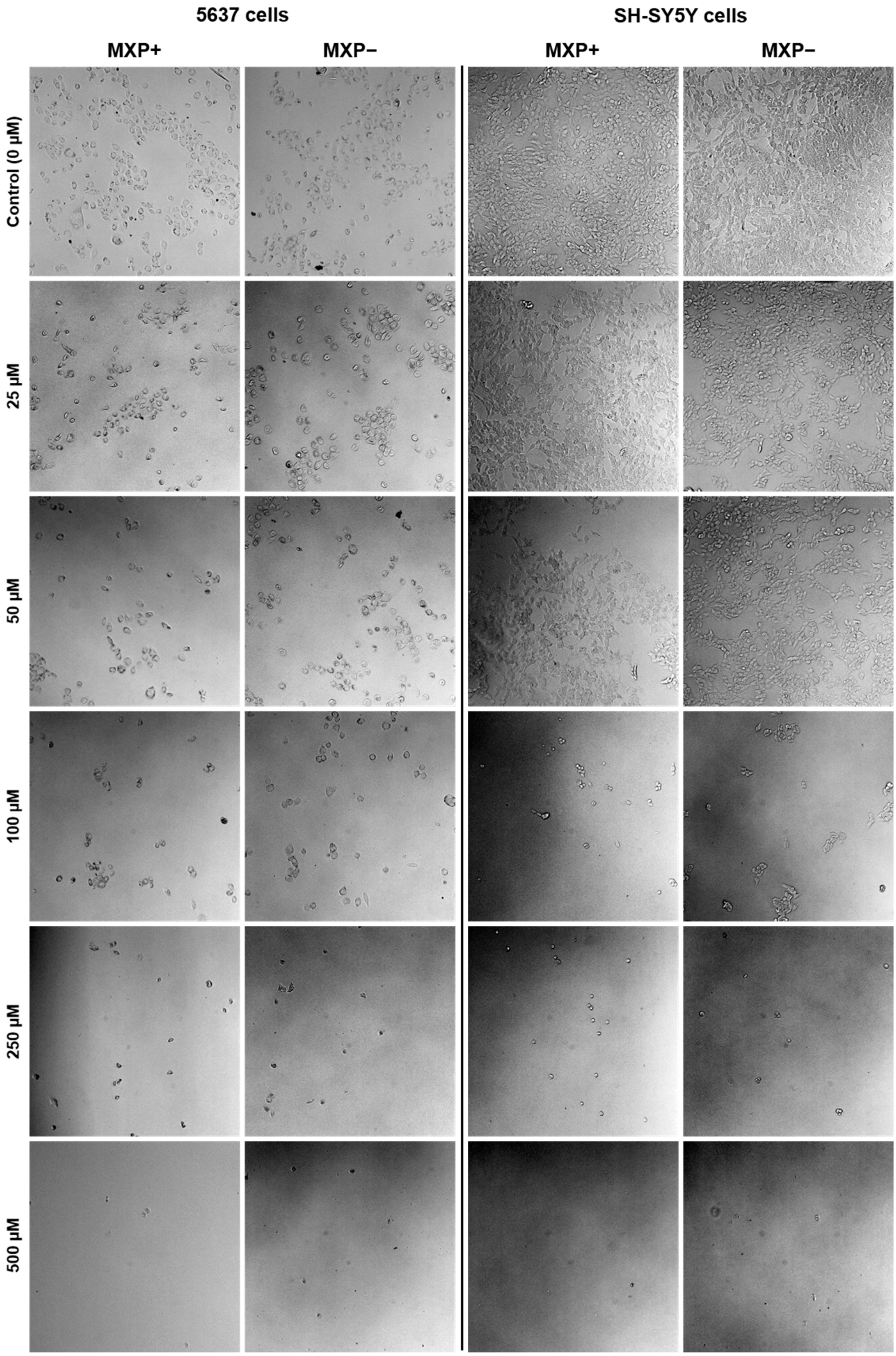

Figure S1. Bright field images of human cells derived from urinary bladder carcinoma (5637) and neuroblastoma (SH-SY5Y) treated with a street sample of methoxphenidine (MXP) containing inorganic impurities (MXP+) and its synthetic standard (MXP−). The 5637 and SH-SY5Y cells were treated with a 0-500-μM concentration of MXP for 72 h. First and third columns represent cells treated with MXP+, the second and fourth columns cells treated with MXP−.
